# Ultrafast multi-focus 3-D nano-fabrication based on two-photon polymerization

**DOI:** 10.1038/s41467-019-10249-2

**Published:** 2019-05-16

**Authors:** Qiang Geng, Dien Wang, Pengfei Chen, Shih-Chi Chen

**Affiliations:** Department of Mechanical and Automation Engineering, The Chinese University of Hong Kong, Shatin, Hong Kong

**Keywords:** Mechanical engineering, Design, synthesis and processing, Lithography

## Abstract

Two-photon polymerization (TPP) is the most precise 3-D printing process that has been used to create many complex structures for advanced photonic and nanoscale applications. However, to date the technology still remains a laboratory tool due to its high operation cost and limited fabrication rate, i.e., serial laser scanning process. Here we present a revolutionary laser nanofabrication process based on TPP and an ultrafast random-access digital micromirror device (DMD) scanner. By exploiting binary holography, the DMD scanner can simultaneously generate and individually control one to tens of laser foci for parallel nanofabrication at 22.7 kHz. Complex 3-D trusses and woodpile structures have been fabricated via single or multi-focus processes, showing a resolution of ~500 nm. The nanofabrication system may be used for largescale nano-prototyping or creation of complex structures, e.g., overhanging structures, that cannot be easily fabricated via conventional raster-scanning-based systems, bringing significant impact to the world of nanomanufacturing.

## Abstract (web summary)

Two photon polymerization (TPP) allows nanofabrication of three dimensional objects with complex geometries, but is considered to be slow with a limited fabrication rate. Here the authors present a TPP technique based on a digital mirror device scanner which allows for fast parallel nanofabrication with improved precision and flexibility.

## Introduction

3-D printing, i.e., additive manufacturing, is one of the most important technological innovation in the past few decades. Two-photon polymerization (TPP) is the 3-D printing process that achieves the highest resolution and has been used to create many complex structures for advanced photonic and nanoscale applications. The application of the two-photon absorption (TPA) phenomenon for micro-fabrication was first proposed and realized in the 1990s^1–4^. By exploiting TPA, TPP fabrication can be achieved with a femtosecond laser in combination with a pair of laser scanners, e.g., galvanometric scanners. During the TPP process, the laser is focused to a highly confined region within a photosensitive resin which induces nonlinear absorption, i.e., two or more photons are simultaneously absorbed by the polymers, and subsequently polymerizes (solidifies) the local resin as nanoscale building blocks, i.e., voxels. Accordingly, 3-D structures can be fabricated by precisely overlapping the voxels via scanning the laser focus or the sample. In recent years, the TPP process has been extensively used to fabricate many micro-structures and nano-structures for a wide range of applications, e.g., photonic crystals or microfluidic devices^5^. The TPP process has a reported lateral resolution of 100 nm and axial resolution of 300 nm^6–8^. Although sub-100 nm resolution has been demonstrated by fine-tuning the laser intensity to the threshold value, the throughput and pattern reproducibility are compromised under these conditions. Recently, TPP fabrication has been commercialized by Nanoscribe GmbH, which reliably achieves a lateral resolution of 400 nm and axial resolution of 1000 nm^9^.

A typical TPP fabrication system includes two galvanometric scanners that raster-scan the laser focus in the *X*-*Y* plane, and a precision *XYZ* stage that performs axial scanning (speed ~ 0.1 Hz) or sample maneuvering. During the printing process, structures are fabricated in a layer-by-layer fashion, i.e., after the completion of one layer, the sample stage moves the build plane axially to polymerize the next layer. Although this process can produce high-resolution 3-D structures, the throughput is limited by the sequential laser scanning process. The disadvantage is more pronounced when printing complex hollow structures, e.g., octet truss^10^, or nonplanar structures as the laser is always required to scan through the entire build volume.

Recently, several parallel TPP methods have been proposed to improve the fabrication throughput^11–17^. For example, by including a microlens array or liquid-crystal spatial light modulator in the light path, one may split the laser focus into multiple foci for parallel processing^11–15^. By spatially and temporally focusing a femtosecond laser in combination with a photomask or digital micromirror device, a patterned light sheet can be generated for laser micro-processing^16,17^. However, these methods are either limited to fabricate periodic structures or compromise fabrication resolution. A high precision, high throughput method for TPP fabrication has yet to be developed.

In this paper, we present a revolutionary laser nanofabrication process based on TPP and an ultrafast random-access digital micromirror device (DMD) scanner. By exploiting binary holography, the DMD scanner can simultaneously generate and control one to tens of laser foci for parallel nanofabrication at the DMD pattern rate, i.e., 22.7 kHz. The axial and lateral scanning resolutions (i.e., minimum step size) are 270 nm and 130 nm, respectively. Previously, we have demonstrated high-speed femtosecond laser beam shaping and single-focus random-access scanning, i.e., the laser focus can move to any random point in the work space at equal speed, using a DMD for optical imaging applications^18–21^. This work is built upon the previous work, which extends the single-focus scanning capability to multi-focus scanning for parallel nanofabrication. As the control of focus position and laser dosage is entirely discretized, the fabrication throughput, grayscale control, precision, and repeatability are substantially improved.

## Results

### Optical design

**Fig. 1:**
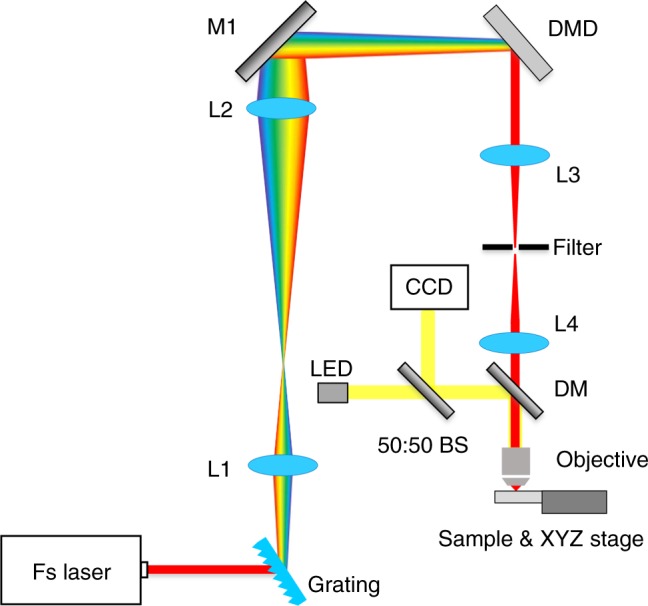
Optical configuration of the TPP fabrication system. Femtosecond laser beams are scanned by the DMD multi-point random-access scanner. M1, high-reflectivity mirrors; DM, dichroic mirror; BS, beam splitter; L1–L4: lenses (*f*_L1_, *f*_L2_, *f*_L3_, *f*_L4_ = 100, 250, 200, 200 mm, respectively)

Figure 1 presents the optical design for the DMD-based nanofabrication system. Firstly, femtosecond laser pulses are generated by a tunable Ti:sapphire laser (680–1080 nm, 200 fs, 3.3 W, Chameleon Ultra II, Coherent), where the laser is set at its central wavelength (i.e., 800 nm) for all TPP fabrication experiments. To fully utilize all pixels on the DMD (DLP 4100 0.7″ XGA, 1024 × 768 pixels, Texas Instrument), L1 and L2 which form a 4-f system adjust the beam diameter to be larger than the DMD aperture. Note that due to the small pixel sizes on the DMD (i.e., 13.68 µm), the device will introduce negative angular dispersion to the laser beam, i.e., laser pulses will be widened and the lowered peak power cannot generate TPA. To compensate angular dispersion, we insert a blazed transmission grating and a high reflectivity mirror (M1) in the optical system to generate positive angular dispersion. (See dispersion compensation design in “Methods” section.) To control the generated laser foci in the focal region of the objective lens, the DMD is placed at the conjugation plane of the objective lens’ back aperture via another 4-f system, i.e., an achromatic lens L3 and a tube lens L4. Lastly, a spatial filter is placed at the back focal plane of L3 to spatially select the 1st order diffraction of the binary hologram. To monitor the nanofabrication process in situ, a microscopic imaging system is built in conjunction with the fabrication setup. As shown in Fig. 1, the microscope shares the objective with the fabrication system. An epi-illumination light source is coupled into the system for sample illumination; the CCD camera after the dichroic mirror records the fabrication process. The sample (i.e., photosensitive resin) is mounted on a motorized precision *XYZ* stage. The resin used in the TPP experiments is IP-Dip (Nanoscribe GmbH). A high NA oil immersion objective (Nikon CFI S Fluor 40× Oil, NA = 1.3, WD = 0.22 mm) is used to match the refractive index of the resin (~1.52).

### Synthesis of binary holograms for single-focus and multi-focus 3-D scanning

**Fig. 2:**
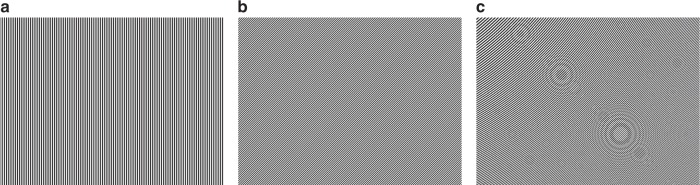
Computer generated holograms based on binary holography. **a** hologram for *x*-axis scanning; **b** hologram for in-plane *x*-*y* scanning; **c** hologram with titled phase and spherical wavefront for 3-D random-access scanning

Manipulation of the dispersion-free laser beam is achieved by applying binary holography (i.e., Lee holography)^18,22^ to a DMD. Equation (1) presents the governing equation of binary holography. To implement it on a DMD, let *A(x,y) ∙* exp[*iφ(x,y)*] be the target wavefront, where *A(x,y)* ∈ [0,1] and *φ(x,y)* represent the amplitude and phase of the electric field respectively; *x* and *y* are the coordinates in the Cartesian coordinate system. Next, let *h(i,j)* ∈ {0,1}, (1 ≤ *i* ≤ *m*, 1 ≤ *j* *≤* *n*, *i, j* ∈ *N*) be the pixels on the DMD, where 1 and 0 refer to the “on” and “off” states respectively; and *m* and *n* refer to the number of rows and columns respectively, i.e., *x* = *i∙d*_D_ and *y* = *j∙d*_D_. Figure 2 presents representative binary holograms generated via Eq. (1).

1 $$h\left( {i,j} \right) = \left\{ \begin{array}{l} 1, - \frac{\sin ^{ - 1}A\left( {x,y} \right)}{2\pi} \le \frac{R\left( {x,y} \right)}{T} + \frac{\varphi \left( {x,y} \right)}{2\pi } + k \le \frac{\sin ^{ - 1}A(x,y)}{2\pi} \\ {0,} \,\, {\mathrm{otherwise}} \end{array} \right.,$$

Single-focus random-access scanning can be achieved by superposing lateral and axial scanning holograms, as shown in Fig. 2c, and rapidly modulating them in the DMD. Lateral scanning can be achieved by modulating the tilted phase term, *R(x,y)*, in Eq. (1), which controls the spatial separation of different diffraction orders; *T* is the grating period and *k* is an integer. Generally, the tilted phase is given by *R(x,y)* = cos*θ∙x* + sin*θ∙y*, where *θ* is the angle of the fringes measured counter-clockwise from the y-axis. Accordingly, by controlling *T* and *θ*, as illustrated in Fig. 2a, b, the first diffraction order can be shifted laterally^21^. Axial scanning can be achieved by applying binary holograms of spherical wavefronts of increasing or decreasing focal lengths to the DMD. The spherical wavefronts are mathematically expressed in Eq. (2)^19^.

2 $$\varphi \left( {x,y} \right) = \frac{{\pi (x^2 + y^2)}}{{\lambda f}},$$

Multi-focus random-access scanning can be achieved by superposing designed binary holograms, where each constituent hologram contains the spatial and intensity information of a designed laser focus. This is feasible as the target wavefront *A(x,y)*∙exp[*iφ(x,y)*] can contain many frequency components instead of one. As such, all focal points generated by the hologram can be independently controlled and arbitrarily positioned in the same work space. Through the intensity distribution control among the focal points, single exposure grayscale control can be realized. To mathematically realize multi-focus scanning, let *A(x,y)*∙exp[*iφ(x,y)*] be the target wavefront containing *k* focal points. Binary holograms with the desired intensity distribution among the *k* focal points can be synthesized via Eq. (3) below:

3 $$h\left( {i,j} \right) = \left\{ \begin{array}{l} 1,\, - A(x,y) \le {\mathop {\sum }\limits_{k = 1}^{n}} B_k\sin \left( 2\pi \frac{R_k\left( {x,y} \right)}{T_{k}} + \varphi _k\left( {x,y} \right) + \varphi _{w,k}\left( {x,y} \right) \right) \le A\left( {x,y} \right) \\ {0,} \,\, {\mathrm{otherwise}}\end{array} \right.,$$

where *h(i, j)* represents the binary value of the micromirrors on the DMD; *B*_*k*_, *R*_*k*_(*x*,*y*), *T*_*k*_, and *ϕ*_*k*_ are the relative amplitude factor, tilted phase, grating period, and phase for the *k*th focal point respectively. *ϕ*_*w,k*_ is the additional wavefront information to be included in the hologram for controlling the size and shape of the focal points. Note that the power of each focal length can be individually controlled by adjusting *B*_*k*_. In practical applications, the number of laser foci is often limited by the total laser power, i.e., each laser focus must have high enough intensity to induce multi-photon adsorption. On the other hand, due to the limited total DMD pixels, increasing the number of laser foci may slightly compromise the print resolution. To optimize the modulation resolution for multiple focal points, iterative Gerchberg-Saxton algorithm^23^ may be applied to design the binary holograms. (Note that the use of Gerchberg-Saxton algorithm may increase the required computational power.)

The relationship between the scanning performance, i.e., range and resolution, and the DMD parameters, i.e., pixel size (*d*_D_) and aperture sizes (~*nd*_D_), has been studied and reported in^19–21^. The results are applicable to both single-focus and multi-focus scanning processes. Briefly, for lateral scanning, the range is inversely proportional to *d*_D_ and the magnification of the objective lens (*M*_obj_); the minimum step size is inversely proportional to *nd*_D_ and *M*_obj_. For axial scanning, the range is inversely proportional to *d*_*D*_^2^ and *M*_obj_^2^; the minimum step size is inversely proportional to *n*^*2*^*d*_*D*_^*2*^ *and M*_obj_^*2*^. When using a 40× objective lens, the work volume of the DMD scanner is calculated to be 103 × 206 × 524 µm^3^; and the scanning resolution is 130 nm and 270 nm in the lateral and axial directions, respectively. As the scanning resolution is much smaller than a voxel, the DMD scanner may be used to realize high-resolution and high-reproducibility TPP fabrication. This is because although the scanning path is entirely discrete and non-continuous, the small step sizes ensure each voxel can be precisely overlapped. Importantly, the control of laser dosage is also discrete, and the smallest pixel dwell time is 44 µs. When used in combination with an electro-optic modulator and the hologram-enabled intensity distribution control, precise dosage and gray scale control can be achieved. Note that when a DMD of higher resolution is used, e.g., DLP6500 (*d*_D_ = 7.68 µm), the work volume will be increased by a factor of 10.07.

### Experiments of the nanofabrication system

**Fig. 3:**
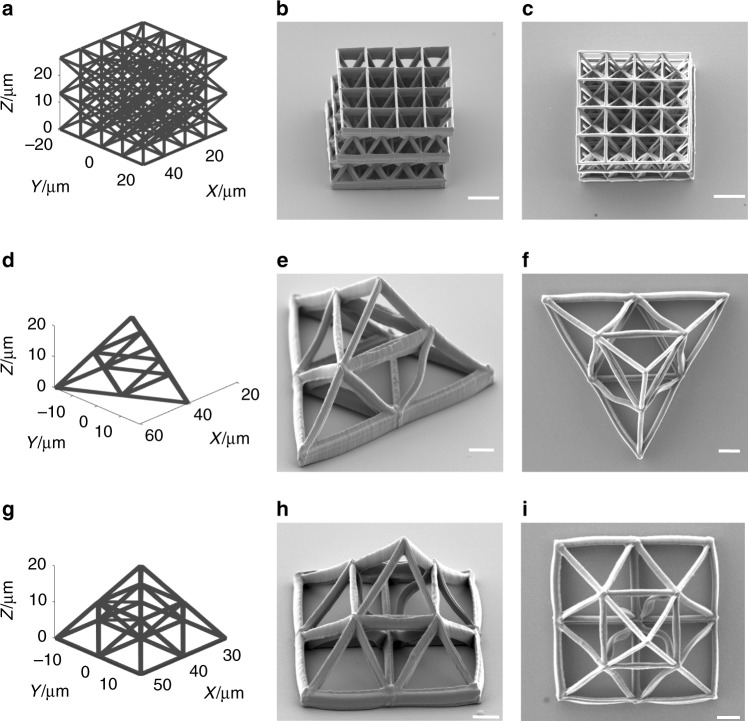
Single-focus DMD fabrication results. **a**–**c** Octet truss, scale bars: 10 µm; **d**–**f** triangular truss, scale bars: 5 µm; **g**–**i** pyramid truss, scale bars: 5 µm

In this section, TPP fabrication experiments are devised and performed to demonstrate the precision and speed of the random-access DMD-scanner. In the first experiment, we fabricate selected truss structures via single-focus writing. The fabrication results are presented in Fig. 3, including octet truss, Fig. 3a–c, triangular truss, Fig. 3d–f, and pyramidal truss, Fig. 3g–i. In these experiments, the laser power is set to be 10 mW with a writing speed of 2 kHz (~0.5 mm/s). From the SEM images, one may observe the fabricated structures match well with the 3-D models with high accuracy and minimal distortion. It is worthwhile to note that during the fabrication process, the laser focus only need to scan through the spaces with designed structures; as such, the total fabrication time of the DMD system is only proportional to the total solid volume regardless of the level of complexity. This is because the DMD can randomly access any point in space with equal speed (up to 22.7 kHz or 5 mm/s), presenting great advantages for fabricating complex structures. In addition, planning of different writing trajectories only requires reordering the holograms stored in the DMD memory. For example, the octet truss structure, shown in Fig. 3(a), consists of ~60,000 points (or holograms), and the total fabrication time is ~60,000/2 kHz = 30 s. At 22.7 kHz, the total fabrication time will be ~2.64 s, with slightly compromised structure resolution due to the increased writing laser intensity^5^.

**Fig. 4:**
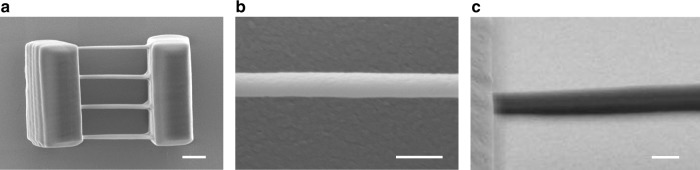
Suspended single-voxel bridges. **a** TPP printed four-bridge structure, scale bar: 3 µm; **b**, **c** zoom-in top view and isometric view of a single bridge in **a**. Scale bars: 1 µm

To investigate the print resolution, four suspended bridges with widths of a single voxel are fabricated via single-focus writing, as shown in Fig. 4a. Figure 4b, c present zoom-in top and isometric views of the bridge, showing a lateral and axial resolution of ~500 nm and ~1600 nm, respectively, which are close to the diffraction limit. Better resolution may be achieved via precise laser intensity control. Note that laser intensity can be directly controlled by the DMD scanner via modulating the amplitude factor *A(x,y)* in Eq. (1). See Supplementary Movie 1 for a demonstration of precise intensity and grayscale control, a checkerboard pattern (40 × 40 µm^2^) is fabricated with a pitch of 2 µm, where the graded structures are directly fabricated without compromising the speed (~5 mm/s; 22.7 kHz) and precision (Supplementary Movie 1). The laser power at the high and low positions alternates between 16 mW and 8 mW, repeatedly; the total write time is ~1.1 s.

**Fig. 5:**
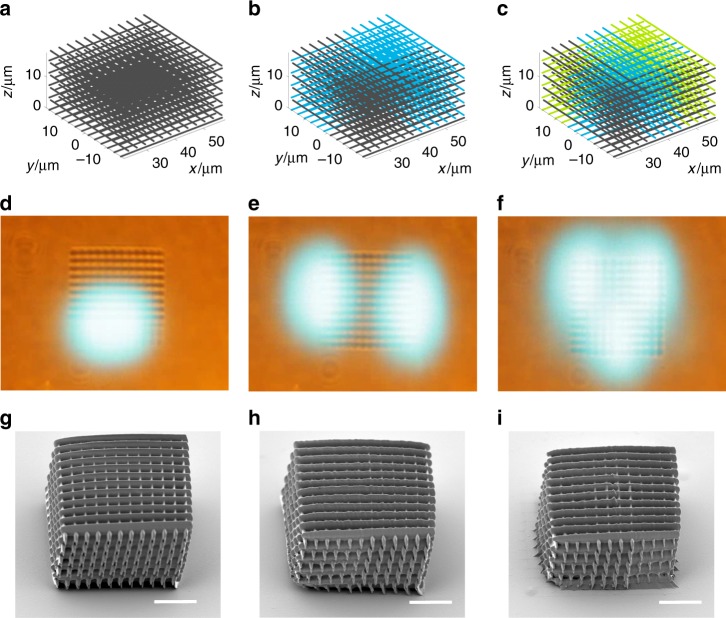
Single-focus, two-focus, and three-focus fabrication results. **a**–**c** planned laser scanning trajectories; **d**–**f** snapshots of the multi-focus TPP fabrication process; **g**–**i** SEM images of the fabricated woodpile structures. Scale bars: 10 µm

Next, we demonstrate the multi-focus TPP fabrication process and compare the results with single-focus fabrication. In the experiments, the DMD scanner is programmed to fabricate a 10-layer woodpile structure (36 × 36 × 20 μm^3^) via the single-focus, two-focus, and three-focus writing mode. In the experiments, the laser power of the single-focus, two-focus, and three-focus mode is set to 10, 20, and 30 mW, respectively. (Note all focal points have equal laser power.) The total number of holograms used for the single-focus, two-focus, and three-focus mode are 21,600, 10,800 and 7200, respectively. The laser scans at 2 kHz and thus the total fabrication time for each mode is 10.8, 5.4, and 3.6 s, respectively. Figure 5a–c present the planned scanning trajectories, where trajectories of different laser foci are labeled by different colors. Figure 5d–f present snapshots of the single-focus, two-focus, and three-focus fabrication processes, respectively, recorded by the built-in microscope (Supplementary Movie 2). Figure 5g–i present the SEM images of the fabrication results via the single-focus, two-focus, and three-focus writing processes, respectively; from the SEM images, one may find all fabricated structures have good resolution and integrity. Further examining the SEM images, it is observed that the single-focus written structures show better surface smoothness. From the experiments, we can confirm that the print resolution is approximately 500 nm (limited by diffraction) for all writing modes.

**Fig. 6:**
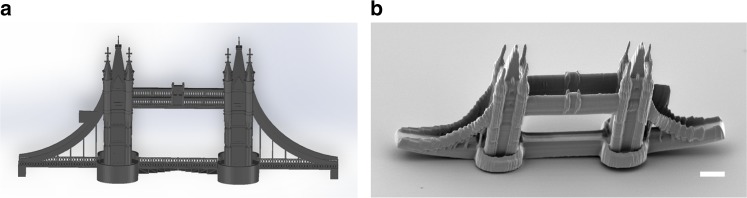
Fabrication of arbitrary structures. **a** CAD model of the microscale London Bridge; **b** SEM image of the TPP fabricated London Bridge. Scale bar: 10 µm

Lastly, we fabricated a microscale London Bridge (120 × 14 × 60 μm^3^) via the DMD scanner, demonstrating the capability of writing arbitrary microstructures. The CAD model and fabrication results are presented in Fig. 6a, b, respectively. To perform the fabrication, the CAD model (in STL format) is first converted into 3-D point arrays with approximately 80,000 points; each point in space is then converted into a unique binary hologram. This process is automated via our custom-developed software. Next, the scanning trajectories are planned by ordering the sequence of the binary holograms. (Note that the total fabrication time remains constant regardless of the writing sequence.) To save fabrication time, holograms can be superposed to perform multi-focus fabrication.

## Discussion

In summary, we have presented a nanofabrication platform for TPP based on the random-access DMD scanner, achieving diffraction-limited fabrication resolution and a writing speed of 22.7 kHz (*n* × 5 mm/s; *n* = number of foci)—the highest fabrication speed reported to date for TPP fabrication, without compromising the resolution. By controlling the amplitude and phase of the input laser via binary holograms and the fast-switching micromirrors, the laser beam can be split into multiple focal points for simultaneous nano-writing. Parametric models for single-focus and multi-focus hologram generation and laser scanning have been developed and verified via nanofabrication experiments, including different truss structures, woodpiles, and a London Bridge. Comparing with existing point-scanning-based solutions, the DMD system presents distinctive advantages in precisely controlling the focus position (~100 nm) and laser dosage (i.e., grayscale control), thereby enabling the design and creation of complex 3-D photonic structures, topologically optimized mechanical devices, and many other structures, e.g., overhanging structures, that are difficult to fabricate through conventional raster-scanning-based systems, bringing significant impact to the world of nanomanufacturing.

## Methods

### Dispersion compensation

In this section, we present a method to fully compensate the angular dispersion introduced by the DMD, which is more accurate and robust compared to the method reported previously^19–21^. The optimal system parameters will be mathematically determined. We begin with the grating equation as expressed in Eq. (4).

4 $$d\left( {\sin \theta _i + \sin \theta _m} \right) = m\lambda ,$$

where *d* is grating period; *m* is an integer that indicates the diffraction order; *θ*_*i*_ and *θ*_*m*_ are the incident and diffraction angles of the *m*th diffraction order respectively. Differentiating Eq. (4), we can obtain the angular dispersion ∂*θ*_*m*_/∂*λ* = *m*/(*d*∙cos*θ*_*m*_). By comparing the angular dispersion between the grating and the DMD, the ratio of the cone angles Δ*θ*_G_ and Δ*θ*_D_ can be obtained and expressed as

5 $$\frac{{\Delta \theta _{\mathrm{G}}}}{{\Delta \theta _{\mathrm{D}}}} = \frac{{m_{\mathrm{G}}d_{\mathrm{D}}\cos \theta _{{\mathrm{iD}}}}}{{m_{\mathrm{D}}d_{\mathrm{G}}\cos \theta _{{\mathrm{mG}}}}},$$

where the subscripts G and D denote the related parameters of the grating and DMD respectively. As shown in Fig. 1, L1 and L2 form a 4-f system that expands the laser beam and adjusts the dispersion angle after the grating. Accordingly, the angular dispersion introduced by DMD can be entirely compensated. As Δ*θ*_G_ and Δ*θ*_D_ are small, they can be approximated as

6 $$\frac{{\Delta \theta _{\mathrm{G}}}}{{\Delta \theta _{\mathrm{D}}}} \approx \frac{{f_{{\mathrm{L2}}}}}{{f_{{\mathrm{L1}}}}},$$

To find the optimal parameters, consider the incident laser beam to the DMD is 45°, the effective DMD pixel size (*d*_D,E_) can be calculated as 13.68/cos(45°) = 19.35 μm; the pitch of the grating (*d*_G_) is 0.83 μm (1200 lines/mm, LightSmyth); the relevant diffraction orders of the grating (*m*_G_) and the DMD (*m*_D_) are 1 and 10, respectively; and the corresponding diffraction angles are *θ*_mG_ = 27° and *θ*_iD_ = 17°, respectively. Substituting these values into Eqs. 5 and 6, we find that *f*_L2_ = 2.5∙ *f*_L1_; accordingly, *f*_L1_ and *f*_L2_ are selected to be 100 mm and 250 mm, respectively. Note that when a different grating or DMD model is used, a suitable 4-f system can always be found to compensate the angular dispersion as *θ*_iD_ can be adjusted continuously in the system.

## Supplementary information

Description of Additional Supplementary Files

Supplementary Movie 1

Supplementary Movie 2

## Data Availability

All data are included in this article and the supplementary movies. Additional information is available on request from the corresponding author (S.C.).

## Code availability

The computer codes that automatically generate binary holograms are available on request from the corresponding author (S.C.)
