# Supplementary material for: Ultrafast multi-focus 3-D nano-fabrication based on two-photon polymerization
Source: Nat Commun. 2019 May 16;10:2179. doi: 10.1038/s41467-019-10249-2 (PMC6522551; doi:10.1038/s41467-019-10249-2)
Supplement: Supplementary file 1 — Description of Additional Supplementary Files [file 41467_2019_10249_MOESM1_ESM.pdf]

Description of Additional Supplementary Files

File name: Supplementary Movie 1

Description: Demonstration of precise intensity and grayscale control. A checkerboard pattern (40 × 40 µm2) is fabricated with a pitch of 2 µm, where the graded structures are directly fabricated without compromising the speed (~5 mm/s; 22.7 kHz) and precision. The laser power at the high and low positions alternates between 16 mW and 8 mW repeatedly; the total write time is ~1.1 s.

File name: Supplementary Movie 2

Description: Demonstration of multi-focus fabrication processes and results. One, two, three and four laser foci have been generated to parallelly write woodpile structures, respectively.
